# Therapeutic potential of traditional Chinese medicine for hyperuricemia: mechanistic insights and clinical prospects

**DOI:** 10.3389/fphar.2026.1862880

**Published:** 2026-08-05

**Authors:** Hong Li, Siqi You, Xiaoping Luo, Yi Huang, Mian Li, Jie Zhou, Sichong Ren

**Affiliations:** 1 Department of Nephrology, School of Clinical Medicine and The First Affiliated Hospital of Chengdu Medical College, Chengdu, China; 2 TCM Preventative Treatment Research Center of Chengdu Medical College, Chengdu, China; 3 The First Affiliated Hospital of Traditional Chinese Medicine of Chengdu Medical College, Xindu Hospital of Traditional Chinese Medicine, Chengdu, China

**Keywords:** gut microbiota, hyperuricemia, multi-target mechanisms, traditional Chinese medicine, urate transporters

## Abstract

Hyperuricemia (HUA) is a common metabolic disorder with rising global prevalence and is closely linked to gout, chronic kidney disease, and cardiovascular complications. Current Western drugs are limited by single-target mechanisms and adverse effects, whereas traditional Chinese medicine (TCM) is proposed to offer multi-component and multi-target therapeutic effects. This review critically synthesizes recent advances in TCM for HUA, focusing on how natural products modulate uric acid production (XOD/ADA), renal/intestinal excretion (URAT1, GLUT9, ABCG2, OATs), inflammation (NLRP3/NF-κB), and gut microbiota via the gut–kidney axis. We propose a four-tier hierarchical framework—purified metabolites, extracts, single botanical drugs, and formulas—that bridges molecular precision with systemic effects: metabolites serve as mechanistic probes, extracts and single botanical drugs achieve multi-component effects, and formulas enable network regulation. This framework helps deconstruct complex TCM interventions into testable, multi-target strategies. Beyond summarizing mechanisms, we critically examine methodological flaws in preclinical studies (acute models, prophylactic dosing, intraperitoneal injection) that limit translational validity, and highlight the pervasive gap between correlative findings (e.g., transporter expression, microbiota shifts) and causal evidence. We also address the under-appreciated safety profile of anti-HUA TCM, including potential organ toxicity and herb–drug interactions. By critically examining limitations, this review aims to provide a realistic, systems-level perspective to guide future mechanistic and translational research.

## 1 Introduction

Hyperuricemia is defined as fasting serum urate levels exceeding 420 μmol/L in men and 360 μmol/L in women on at least two separate occasions under a normal purine diet. Its pathophysiology primarily involves dysregulated purine metabolism and/or impaired renal and intestinal uric acid excretion (Mandal and Mount, 2015). Driven by dietary shifts, sedentary lifestyles, genetic predisposition, and population aging, the global burden of hyperuricemia has risen dramatically, becoming a major public health challenge (Asghari et al., 2024). Importantly, hyperuricemia is not merely a biochemical abnormality but a critical risk factor for multiple comorbidities, including gout, chronic kidney disease (CKD), cardiovascular disease (CVD), hypertension, type 2 diabetes mellitus (T2DM), and metabolic syndrome (Hisatome, 2024; Kuwabara et al., 2023).

As hyperuricemia progresses, monosodium urate (MSU) crystals may deposit in joints and soft tissues, triggering inflammatory cascades that lead to acute gouty arthritis and long-term joint damage, while also promoting renal inflammation and interstitial fibrosis (Li et al., 2023). Epidemiological studies further indicate that hyperuricemia prevalence is rising not only in Western countries (Carter and Roman, 2025) but also across Asian populations, including China, Japan, and South Korea, underscoring the urgent need for effective and safe therapeutic strategies (Ahn, 2023; Aoki et al., 2022; Zhang et al., 2021).

Conventional pharmacological management of hyperuricemia primarily relies on xanthine oxidase (XO) inhibitors (e.g., allopurinol, febuxostat) to reduce uric acid production, or uricosuric agents (e.g., probenecid, benzbromarone) to enhance renal excretion. However, long-term use of these agents is frequently limited by adverse effects, including hepatotoxicity (Li et al., 2025), hypersensitivity reactions (Stamp et al. 2016), and nephrotoxicity (Helget et al., 2024), which adversely affect patient adherence and compromise sustained outcomes. For instance, allopurinol may induce hypersensitivity syndrome in up to 2% of users, particularly among HLA-B5801 carriers (Ahn et al., 2025; Yaseen et al., 2023), while febuxostat has been associated with increased cardiovascular risk according to FDA warnings (White et al., 2018). Uricosuric agents may elevate nephrolithiasis risk or interact with concomitant medications, resulting in long-term adherence rates of only ∼50% in clinical studies (Yin et al., 2018).

Moreover, these monotherapies typically address only a single node of the complex uric acid metabolic network and fail to tackle the systemic nature of hyperuricemia, including its interplay with insulin resistance, dyslipidemia, and gut microbiota dysbiosis. These interrelated factors collectively sustain a vicious cycle of metabolic dysfunction, suggesting that current strategies are insufficient to comprehensively manage hyperuricemia and its associated comorbidities.

Against this background, traditional Chinese medicine (TCM) has emerged as a promising complementary approach to address the limitations of conventional therapies. Grounded in a holistic philosophy, TCM restores systemic balance using complex botanical formulas and natural metabolites. This approach is characterized by multi-target effects, simultaneously modulating multiple biochemical pathways implicated in hyperuricemia (Guo et al., 2025). Importantly, TCM not only lowers serum urate but also corrects underlying metabolic dysfunctions such as insulin resistance, obesity, and chronic low-grade inflammation. The principal anti-hyperuricemic mechanisms of TCM include: (1) inhibition of urate-producing enzymes (xanthine oxidase, XOD; adenosine deaminase, ADA); (2) regulation of renal and intestinal urate transporters (e.g., URAT1, GLUT9, ABCG2, OATs); (3) suppression of inflammatory responses (e.g., NLRP3/NF-κB pathways); (4) attenuation of oxidative stress; and (5) modulation of gut microbiota via the gut–kidney axis.

In this review, we comprehensively summarize recent advances in TCM for hyperuricemia, with a particular focus on how bioactive metabolites interact with key molecular targets involved in urate metabolism, inflammation, and metabolic dysfunction. We also discuss the clinical benefits of TCM-based therapies and their potential for integration into mainstream practice. By bridging traditional theory with modern biomedical research, TCM represents a promising strategy for developing safe and effective therapeutic approaches.

Several recent reviews have addressed TCM in HUA from different perspectives, including systematic evaluation of clinical efficacy (Chen et al., 2020), hyperuricemic nephropathy (Yang et al., 2022), model construction (Zhou et al., 2024), integration with GWAS findings (Yang et al., 2025), and evidence mapping of methodological quality (Li et al., 2026). However, most of these overviews focus on isolated mechanisms, specific categories of botanical drugs, or evidence quality assessment, without fully capturing the multi-target interplay emphasized in this review. The present review distinguishes itself by providing a comprehensive multi-target framework that integrates urate synthesis, transporter regulation, inflammation control, oxidative stress relief, and gut–kidney axis modulation. We organize these mechanisms across four hierarchical levels (metabolites, extracts, single botanical drugs, and formulas) and incorporate the latest evidence, offering an updated and holistic perspective. This hierarchy is not merely categorical but reflects an increasing gradient of biological complexity and therapeutic integration, from a single molecular entity to a polychemical, polypharmacological network. This perspective helps to understand how TCM achieves its systemic effects and provides a roadmap for deconstructing complex formulas into evidence-based lead combinations.

In addition to summarizing the literature, this review briefly discusses the scientific quality of preclinical and clinical studies, noting inconsistencies, knowledge gaps, and common methodological limitations (e.g., lack of blinding, small sample sizes, heterogeneous TCM preparations). These observations may help readers distinguish well-established findings from preliminary evidence and identify areas that warrant further investigation.

## 2 Literature search and study selection

This review adopts a critical narrative approach. To ensure transparency and reproducibility, a structured literature search was performed, with distinct strategies developed for the two thematic components of this review: “epidemiology, diagnosis, and conventional treatment of hyperuricemia” and “mechanisms of uric acid homeostasis regulation by traditional Chinese medicine (TCM)”.

For the epidemiological, diagnostic, and conventional treatment context, targeted, non-systematic searches were conducted in PubMed using relevant keywords (e.g., “hyperuricemia epidemiology,” “gout diagnosis,” “urate-lowering therapy,” “chronic kidney disease hyperuricemia”) to support the background sections, with no strict time restrictions applied.

For TCM mechanism studies, a comprehensive literature search was conducted in the PubMed database using the following search string: (“Hyperuricemia” [Mesh] OR “uric acid”) AND (“Drugs, Chinese Herbal” [Mesh] OR “chinese herb”). The search covered the period from January 2005 to March 2025. This search yielded approximately 300 records. After title and abstract screening against predefined inclusion criteria, approximately 140 records were retained for full-text assessment. Following full-text review, with a preference for studies published after 2010, approximately 80 studies were ultimately included.

Inclusion criteria for TCM mechanism studies were (1) hyperuricemia or related disorders (e.g., gouty arthritis, uric acid nephropathy): as the explicit research subject; (2) studies investigating the mechanisms of action of TCM interventions, including purified metabolites, extracts, single botanical drugs, or formulas; (3) for botanical drugs, the plant species must be clearly identified with full scientific names and, if applicable, listed in classical Chinese pharmacopoeias or modern national pharmacopoeias; (4) peer-reviewed original experimental studies (*in vivo* or *in vitro*), clinical trials, or systematic reviews providing mechanistic data. Exclusion criteria included: (1) studies without original mechanistic data (e.g., pure clinical observations, empirical summaries, or theoretical discussions); (2) small-sample case reports or non-peer-reviewed literature; (3) duplicate publications; and (4) studies focusing on Western pharmacological agents without TCM components.

All retrieved records were independently screened by two authors (HL and SQY) based on titles and abstracts. Any disagreements were resolved through consensus discussion. To minimize the risk of missing relevant studies, the reference lists of all included articles and relevant review papers were manually screened for additional publications. The methodological quality of the selected studies was critically evaluated based on parameters such as model validity (e.g., acute vs. chronic hyperuricemia models), extract characterization, dosing rationale, and the inclusion of appropriate controls, as elaborated in Section 7.1. Since no new experimental characterization of plant materials was performed by the authors, the ConPhyMP checklists are provided in the Supplementary Material and marked as “NOT APPLICABLE”.

## 3 Etiology and pathogenesis of hyperuricemia

Hyperuricemia is a metabolic disorder caused by abnormalities in purine metabolism or impaired uric acid excretion. Its pathogenesis is complex, involving a multifactorial interaction of genetic, environmental, and metabolic factors. Uric acid, as the final product of purine metabolism, is primarily produced endogenously (approximately 80%) with the remaining 20% derived from purine-rich foods such as organ meats and seafood. Under normal physiological conditions, approximately two-thirds of uric acid is excreted via the kidneys, with the remaining one-third excreted through the intestines. The renal excretion of uric acid involves a complex process including glomerular filtration, proximal tubular reabsorption, and secretion. In a healthy state, the production and excretion of uric acid maintain a dynamic balance (Mandal and Mount, 2015). However, disruption of this balance by various factors, including excessive uric acid production or impaired excretion, may lead to the development of hyperuricemia. (Du et al., 2024).

First, abnormalities in purine metabolism are a primary cause of increased uric acid production. Overactivity of purine-metabolizing enzymes, such as XOD (Polito et al., 2022), or genetic mutations (e.g., defects in the HPRT1 gene) (Vinokurov et al., 2023) that lead to increased purine synthesis, result in excessive uric acid production. Additionally, high-purine diets (e.g., organ meats, seafood), alcohol consumption (particularly beer), and fructose intake promote purine breakdown (Zhang et al., 2022), further elevating serum urate levels. Tumor lysis syndrome (Howard et al., 2024), rhabdomyolysis, and other diseases can also accelerate purine metabolism, causing acute elevation in uric acid levels.

Second, approximately 90% of hyperuricemia patients exhibit impaired renal uric acid excretion, which is primarily related to abnormalities in renal tubular urate transporters (So and Thorens, 2010). For instance, overexpression of urate transporter-1 (URAT1) and glucose transporter-9 (GLUT9) increases uric acid reabsorption, while defects in organic anion transporters 1/3 (OAT1/OAT3) or mutations in ATP-binding cassette transporter G2 (ABCG2) reduce uric acid secretion. Furthermore, recent studies have demonstrated that dysbiosis of the gut microbiota may also contribute to elevated serum urate levels. This is likely due to altered microbial metabolism in the intestines, which reduces uric acid excretion through the gut (Du et al., 2024; García-Arroyo et al., 2018; Terkeltaub and Dodd, 2025).

It is noteworthy that contemporary dietary changes, particularly the increased intake of fructose, have become major drivers of the younger onset of hyperuricemia. Fructose specifically activates the ketohexokinase (KHK) pathway, resulting in rapid ATP depletion and consequent AMP accumulation. Excess AMP is then degraded to inosine monophosphate (IMP) and hypoxanthine, which are further oxidized to uric acid by xanthine oxidase. Additionally, lactate, a metabolic product of fructose, competitively inhibits renal tubular uric acid secretion, further exacerbating the elevation of serum urate levels (Lubawy and Formanowicz, 2023; Rho et al., 2011; Zhang et al., 2020).

Moreover, chronic kidney disease, the use of thiazide diuretics, and metabolic acidosis (e.g., lactic acidosis) can all impair uric acid excretion. Genetic factors, such as polymorphisms in the SLC2A9 and SLC22A12 genes (Fujii et al., 2025; Gérard et al., 2025), as well as environmental factors, including obesity, high-fat diets, and psychological stress, may also contribute to the worsening of uric acid metabolic imbalance. Studies have shown that inflammatory cytokines released from adipose tissue in obese individuals can promote uric acid synthesis (Liang et al., 2024), while psychological stress (Ha et al., 2025), through activation of the sympathetic nervous system, further impairs uric acid excretion.

When serum uric acid levels rise, urate crystals and their deposition can lead to multi-organ damage through several pathways. When serum urate concentration exceeds 6.8 mg/dL, monosodium urate crystals precipitate and deposit in the synovial membranes of joints, activating the NLRP3 inflammasome and triggering caspase-1-dependent release of IL-1β. This induces neutrophil chemotaxis, leading to the acute onset of gouty arthritis. Recurrent inflammation can stimulate synovial cell proliferation and cartilage matrix degradation, ultimately leading to tophi formation (Dalbeth et al., 2021; Poulsen and Dalbeth, 2025; Zhang, 2023).

In the kidneys, crystal deposition causes tubular obstruction and interstitial inflammation. Uric acid also activates the renin-angiotensin system, promoting TGF-β1 expression, which leads to interstitial fibrosis and glomerulosclerosis (Zhang et al., 2023). Additionally, when uric acid becomes supersaturated in the urine, it forms urate crystals (Deng et al., 2023), which can obstruct renal tubules or the urinary tract, leading to renal colic and kidney damage (Su et al., 2020).

Notably, in the cardiovascular system, hyperuricemia can impair endothelial function by inhibiting endothelial nitric oxide synthase (eNOS) activity while inducing MCP-1 expression and promoting monocyte infiltration, thus accelerating the progression of atherosclerosis (Nie et al., 2021; Wang et al., 2023; Yazdi et al., 2022).

At the metabolic level, uric acid reduces insulin sensitivity by interfering with the phosphorylation of insulin receptor substrate-1 (IRS-1), thereby inhibiting the AMPK pathway and increasing NLRP3 inflammasome production (Kimura et al., 2020). This induces insulin resistance (Zhao et al., 2022). In adipose tissue, AMPK inhibition together with elevated oxidative stress impairs mitochondrial biogenesis and thermogenic or browning capacity of adipocytes, favoring lipid accumulation and white adipose tissue expansion. These changes are closely linked to the development of systemic insulin resistance. Furthermore, chronic metabolic inflammation induces adipose tissue dysfunction (Dong et al., 2023). Hyperuricemia thus forms a vicious cycle with obesity and dyslipidemia, collectively contributing to the core pathophysiology of metabolic syndrome (Vareldzis et al., 2024). Recent studies have also found that urate crystals can activate microglial cells, promoting β-amyloid deposition, which is associated with the pathological progression of Alzheimer’s disease (Cobo et al., 2022; Venegas et al., 2017).

**FIGURE 1:**
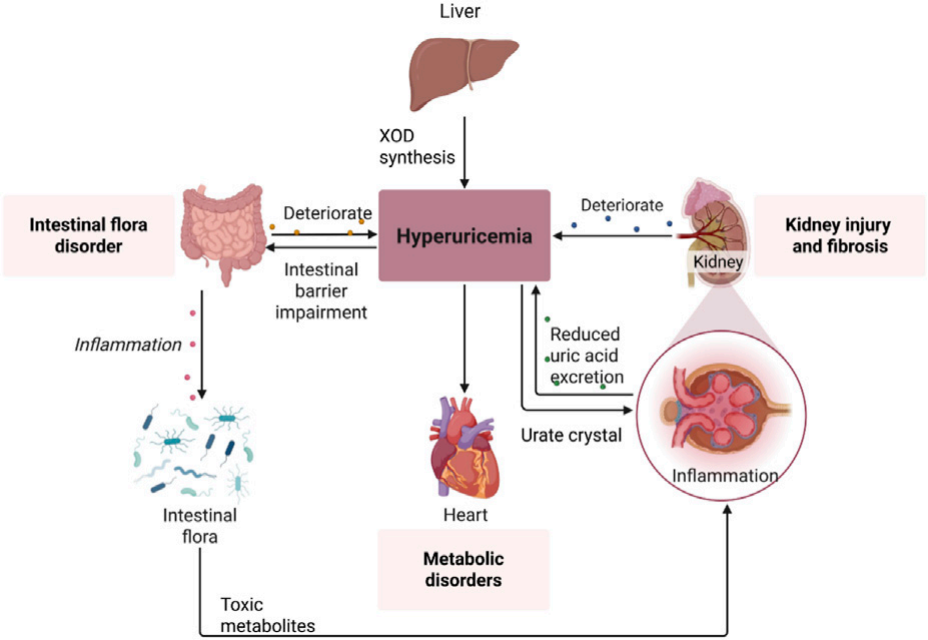
Complications and pathogenesis of hyperuricemia. Hyperuricemia results from increased uric acid production and impaired renal excretion, and is closely associated with gut dysbiosis, inflammation, and multi-organ injury, including kidney, heart, and metabolic disorders. These factors interact to form a vicious cycle that aggravates disease progression. Created in BioRender. LI, H. (2026) https://BioRender.com/5ucku87.

A common mechanism underlying these multi-organ damages is that uric acid disrupts cellular energy metabolism through the AMPK/mTOR pathway (Crişan et al., 2017), compounded by NADPH oxidase-mediated oxidative stress, ultimately leading to cellular apoptosis and organ dysfunction. This cascade of events suggests that early reduction of serum urate levels is not only crucial for preventing gout flares but also serves as a key strategy for blocking systemic complications (Fan et al., 2023; Lu et al., 2020; Wu et al., 2021). The complications and pathogenesis of hyperuricemia are shown in Figure 1.

## 4 Epidemiology of hyperuricemia

In recent years, the prevalence of hyperuricemia has risen rapidly. In the United States, the prevalence of hyperuricemia was reported to be 20.1% in 2015–2016, with an increase of more than 10% over the past decade (Chen-Xu et al., 2019). In China, the prevalence of hyperuricemia has reached 14.0%, with over 180 million affected individuals, making it the fourth most prevalent metabolic disorder after hypertension, hyperglycemia, and hyperlipidemia. Serum urate levels are influenced by multiple factors, including age, sex, race, genetics, dietary habits, medications, and environmental factors. It is primarily observed that men are more affected than women, with higher prevalence rates in urban areas compared to rural regions (Wang et al., 2022), coastal areas compared to inland regions, and a trend toward younger age groups (Liu et al., 2015).

In a nationwide survey on the prevalence of chronic metabolic diseases in ten provinces of China, the prevalence of hyperuricemia increased from 11.39% in 2009 to 14.54% in 2023 (Li et al., 2021). This rise is closely associated with rapid economic development, increased consumption of purine-rich foods (such as seafood, red meat, and alcohol), and greater fructose intake. While hyperuricemia was traditionally considered a condition predominantly affecting middle-aged and elderly men (comprising 95% of cases), a notable trend toward younger ages has emerged in recent years. In particular, the proportion of patients aged under 30 has steadily increased among individuals with hyperuricemia in China (Shi et al., 2023; Wan et al., 2021). Geographically, the prevalence is higher in economically developed southern and coastal regions (such as Guangdong and Zhejiang), which can be attributed to the high consumption of seafood, rich soups, and beer in these areas (Huang et al., 2020; Zhang et al., 2021).

Moreover, sustained elevation of serum urate levels leads to damage in multiple organs. Beyond the occurrence of gout, hyperuricemia is associated with various secondary metabolic diseases. Studies have shown that hyperuricemia is highly comorbid with metabolic syndrome, which includes obesity, hypertension, and diabetes (Yanai et al., 2021). Approximately 20% of hyperuricemia patients also have hypertension (Zhang et al., 2022), and nearly 50% of young individuals with hypertension have concomitant hyperuricemia (Wang, et al., 2017). Elevated serum urate levels are positively correlated with the risk of cardiovascular events and are also significantly associated with Parkinson’s disease (Zhang et al., 2024). Additionally, lifestyle factors such as late-night activities, sedentary behavior, intense physical exercise, and psychological stress exacerbate uric acid metabolic disorders.

Surveys show that approximately one-third of chronic gout patients have renal dysfunction, with chronic urate nephropathy (gouty nephropathy) being the predominant form (Kannuthurai and Gaffo, 2023). This condition is characterized by interstitial fibrosis and renal tubular atrophy. Among patients with stages 3–5 chronic kidney disease, the prevalence of hyperuricemia is as high as 70%–85% (Nie et al., 2025). Evidence from longitudinal cohort studies indicates that each 1 mg/dL (≈60 μmol/L) increment in serum uric acid is associated with an approximately 7% higher risk of progression to kidney failure, supporting the notion that hyperuricemia may serve as a clinically relevant and potentially modifiable risk factor in chronic kidney disease progression (Barman et al., 2023; Tsai et al., 2017).

## 5 Diagnosis and treatment of hyperuricemia and associated disorders

Hyperuricemia is biochemically defined as a sustained elevation of serum urate (SU) above the physiological saturation point for monosodium urate (MSU), approximately 6.8 mg/dL (∼405 μmol/L) at 37 °C, beyond which MSU crystallization and tissue deposition become thermodynamically feasible (Martillo et al., 2014). In practice, operational cutoffs are frequently applied in epidemiologic and clinical contexts, and guideline-based management emphasizes that isolated hyperuricemia is not synonymous with gout; instead, clinical decision-making should integrate symptoms, evidence of urate deposition, and comorbidity profiles (Benn et al., 2018). Accordingly, contemporary recommendations advise a structured assessment of cardiometabolic and renal comorbidities, as these conditions influence both the risk of progression to clinically overt gout and the selection and safety of urate-lowering therapy strategies (FitzGerald et al., 2020).

For gout diagnosis, the identification of MSU crystals in synovial fluid or tophus aspirates remains the diagnostic gold standard. When aspiration is infeasible or nondiagnostic, imaging has an increasingly central role. The 2023 EULAR recommendations on imaging in crystal-induced arthropathies support the use of musculoskeletal ultrasound and dual-energy CT to detect urate deposition and to assist with diagnosis and monitoring in appropriately selected clinical scenarios, thereby improving diagnostic confidence and enabling objective assessment of crystal burden over time (Mandl et al., 2024). A dedicated systematic literature review performed to inform these EULAR imaging recommendations further substantiates the diagnostic and monitoring utility of imaging modalities across crystal-induced arthropathies, including gout (Gessl et al., 2024).

Long-term management has converged toward a treat-to-target paradigm in patients with established gout, aiming to maintain SU below the saturation threshold to promote crystal dissolution and prevent recurrent flares and structural damage. The 2020 American College of Rheumatology (ACR) guideline and the 2016 updated EULAR recommendations both endorse urate-lowering therapy for patients with indications such as frequent flares, tophaceous disease, or radiographic damage, alongside prophylaxis during ULT initiation to reduce flare risk (FitzGerald et al., 2020; Richette et al., 2017). In addition, guidance addressing hyperuricemia in high cardiovascular-risk populations highlights the importance of individualized risk stratification and careful selection of ULT in the setting of cardiovascular comorbidity (Borghi et al., 2024).

Safety considerations are especially salient when choosing between xanthine oxidase inhibitors. The CARES trial reported higher all-cause and cardiovascular mortality with febuxostat compared with allopurinol in gout patients with established cardiovascular disease, whereas the FAST trial—with a different design and population—found febuxostat to be non-inferior to allopurinol regarding major cardiovascular outcomes (Mackenzie et al., 2020; White et al., 2018). Collectively, these findings support a patient-specific approach that incorporates baseline cardiovascular risk, prior drug tolerability, and the feasibility of close follow-up.

Regarding the timing of ULT initiation, evidence syntheses suggest that starting ULT during an acute flare does not clearly worsen pain severity, discontinuation rates, or flare course when appropriate anti-inflammatory management is provided (Tai et al., 2024). This supports guideline strategies that prioritize achieving urate targets without unnecessary delays, while ensuring adequate flare prophylaxis and monitoring.

In patients with chronic kidney disease (CKD), treatment decisions should distinguish between gout indications and attempts to modify CKD progression in asymptomatic hyperuricemia. The KDIGO 2024 CKD guideline does not recommend routine ULT solely to slow CKD progression in patients with asymptomatic hyperuricemia, reflecting limited and inconsistent evidence for renoprotection in this context (KDIGO, 2024). Nonetheless, when gout is present, ULT may still be indicated, with dosing and drug choice tailored to kidney function and adverse-event risk profiles, consistent with multidisciplinary consensus approaches (Chinese Multidisciplinary Expert Consensus on the Diagnosis and Treatment of Hyperuricemia and Related Diseases, 2017). Finally, in patients with overlapping cardiometabolic disease, an integrated strategy that targets urate burden while optimizing cardiovascular and metabolic therapies may provide additive benefit. Empagliflozin demonstrated cardiovascular and mortality benefit in high-risk type 2 diabetes, and SGLT2 inhibitors as a class have been shown to reduce SU modestly in randomized-trial meta-analysis, supporting their preferential use when otherwise clinically indicated (Zhao et al., 2018; Zinman et al., 2015). Similarly, sacubitril/valsartan reduced SU compared with enalapril in PARADIGM-HF, and fenofibrate lowered uric acid and reduced gout events in a *post hoc* analysis of the FIELD study, suggesting that selecting evidence-based cardiometabolic therapies can concurrently contribute to urate control in appropriate patients (Mogensen et al., 2018; Waldman et al., 2018).

## 6 Research progress on traditional Chinese medicine in treating hyperuricemia and its related complication

Modern medicine posits that hyperuricemia results from either excessive uric acid production or impaired renal excretion. While Western pharmacological treatments for hyperuricemia, such as allopurinol, febuxostat, and benzbromarone, are effective in lowering serum uric acid levels, they are not without limitations. These single-agent medications typically target only one aspect of the disease process (e.g., allopurinol inhibits uric acid production, while benzbromarone enhances its excretion). In contrast, TCM offers a more holistic approach by intervening in both the “production-excretion” pathways. Recent advancements in TCM research have highlighted its multi-target capabilities and low toxicity, making it an increasingly prominent area of study. Through multi-target effects, TCM demonstrates distinct advantages while minimizing potential side effects.

In terms of inhibiting uric acid production, several active metabolites derived from botanical drugs have been identified as natural xanthine oxidase inhibitors, effectively reducing uric acid synthesis at the source. This mechanism is comparable to that of allopurinol; however, as these metabolites are naturally derived, their inhibitory effects tend to be milder. On the other hand, TCM excels in promoting uric acid excretion through the regulation of renal urate transporters. Studies have shown that many botanical drugs significantly downregulate the expression of urate reabsorption transporters (such as URAT1) on the apical membrane of renal proximal tubular epithelial cells, while simultaneously upregulating urate excretion transporters (such as OAT1 and ABCG2) on the basolateral membrane. This dual regulation of transporter expression effectively facilitates uric acid excretion via urine.

The multi-target nature of TCM is particularly valuable in the prevention and management of complications associated with hyperuricemia (Wang et al., 2024). Many botanical drugs not only reduce the maturation and release of key inflammatory factors but also downregulate proteins involved in inflammatory signaling pathways, thereby interrupting multiple steps of the inflammatory cascade. This action alleviates the redness, swelling, heat, and pain associated with gout. Furthermore, numerous botanical drugs possess antioxidant properties, which help to neutralize free radicals and mitigate the damage to renal tubular epithelial cells induced by elevated uric acid levels. Additionally, many diuretic and dampness-expelling botanical drugs naturally increase urine output, aiding in the flushing of the urinary tract, preventing the formation of micro-stones, and reducing kidney damage. Recent studies have also highlighted TCM’s significant effects on regulating glucose metabolism, lipid metabolism, and the abundance of gut microbiota. The multi-component, multi-target, and overall regulatory characteristics of TCM provide significant advantages in the treatment of hyperuricemia (Luo et al., 2023).

**FIGURE 2:**
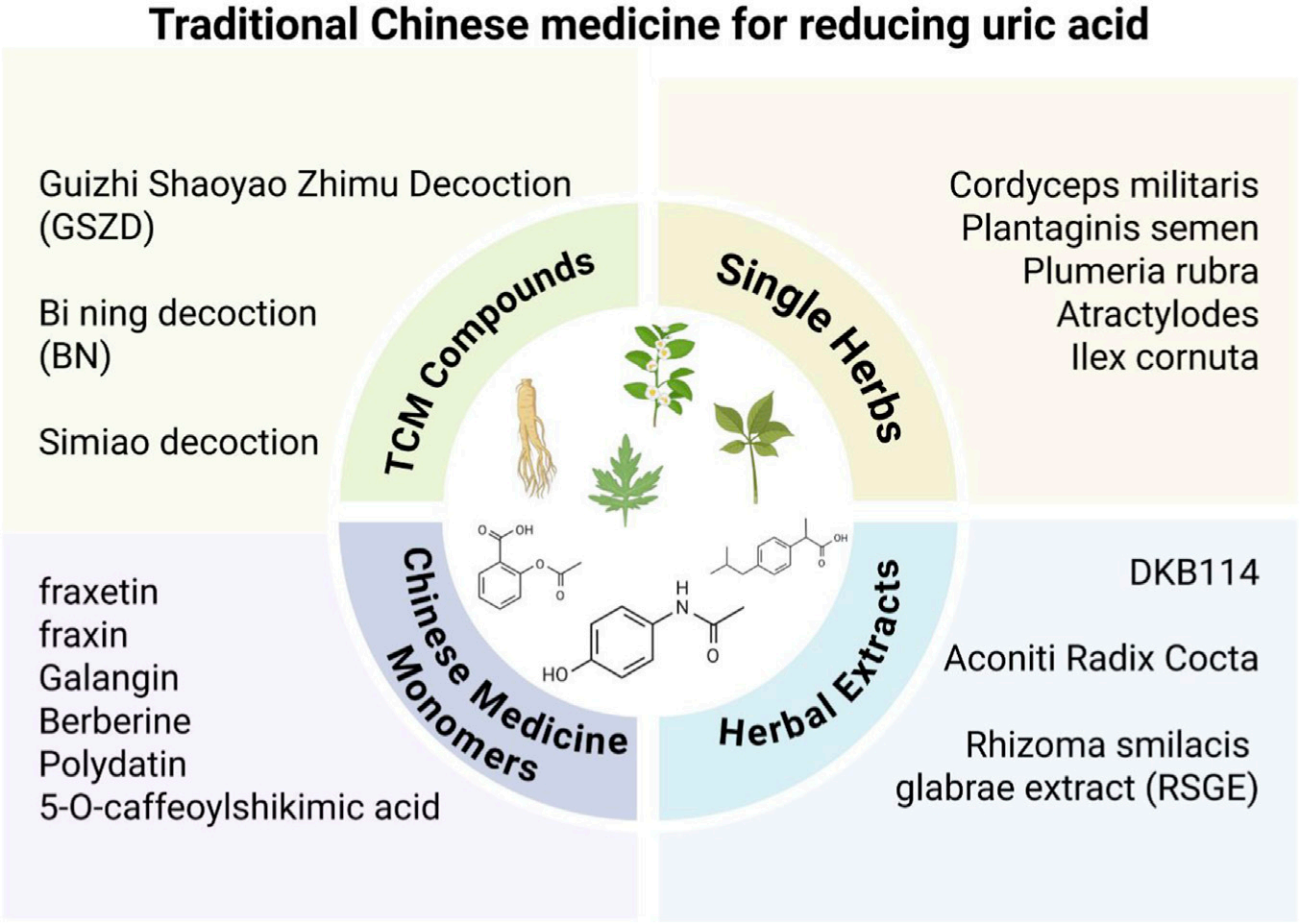
Traditional Chinese medicine for reducing uric acid. Multiple traditional Chinese medicines, including single botanical drugs, purified metabolites, and formulas, reduce serum uric acid levels through multi-component and multi-target mechanisms. Created in BioRender. LI, H. (2026) https://BioRender.com/adl6juf.

Research on TCM for lowering uric acid encompasses studies on single botanical drugs, formulas, and extracts, which exert their effects through anti-inflammatory actions, uric acid-lowering mechanisms, gut microbiota modulation, and renal protection (Liu et al., 2022). These therapeutic effects are mediated through various metabolic pathways and signaling cascades. This review summarizes recent *in vivo* and *in vitro* research that elucidates the ways in which purified metabolites, Extracts, Single botanical drugs, and TCM formulas influence multiple signaling pathways and metabolic processes to improve hyperuricemia and its associated complications. Several traditional Chinese medicines for reducing uric acid are shown in Figure 2.

### 6.1 Regulation of hyperuricemia by purified metabolites and extracts

The purified metabolites and extracts exhibit multi-level and highly specific interventions in regulating the pathological processes of hyperuricemia and its associated complications. This precision is not merely the result of a multi-target effect, but rather reflects the identification and precise regulation of key molecular targets across different pathological stages.

In inhibiting uric acid production, the precision lies in the targeted inhibition of key enzymes in the purine metabolic pathway. For example, purified metabolites such as resveratrol and gallic acid serve as competitive inhibitors of xanthine oxidase (XOD), directly binding to its active site and blocking the catalytic reaction. The regulation by *Astragalus mongholicus* Bunge (Fabaceae) polysaccharides is more systematic, as it not only downregulates the expression of XOD and adenosine deaminase but also precisely upregulates the activity of hypoxanthine-guanine phosphoribosyltransferase (HGPRT), thereby modulating the purine metabolic balance in the liver from both positive and negative directions.

In promoting uric acid excretion, the precision is reflected in the specific regulation of the transmembrane transport protein network. Fraxin, for instance, acts as specific activating ligands for the ABCG2 protein, directly enhancing the function of the renal excretion pump and promoting the active transport of uric acid into the renal tubule lumen. Meanwhile, metabolites such as berberine and isobavachin chalcone selectively downregulate the expression of URAT1 on the apical membrane and GLUT9 on the basolateral membrane of proximal tubular epithelial cells in the kidney, thereby precisely reducing the reabsorption capacity of uric acid with minimal impact on other ion transport pathways.

At the organ protection level, precise regulation focuses on key inflammatory and fibrosis signaling nodes. Resveratrol, for example, effectively inhibits the nuclear translocation of the NF-κB p65 subunit and blocks the assembly of the NLRP3 inflammasome, thereby suppressing the production of core inflammatory mediators such as IL-1β and TNF-α at both the transcriptional and post-translational levels. In the process of renal fibrosis, 5-O-caffeoylshikimic acid specifically downregulates the signaling of transforming growth factor-β1 (TGF-β1) and its downstream Smad proteins, precisely interfering with fibroblast activation and collagen deposition.

Furthermore, certain purified metabolites, such as punicalagin and resveratrol, extend their precise regulation to the intestinal-kidney axis system. Punicalagin reshapes gut microbiota by increasing short-chain fatty acid-producing genera including *Prevotellaceae_UCG-001* and Muribaculaceae, while decreasing pro-inflammatory taxa such as *Parabacteroides*, *Oscillibacter*, *Desulfovibrio* and *Tuzzerella*. Resveratrol enriches multiple *Lactobacillus* species (e.g., *Lactobacillus_*sp._*ESL0791*, *Lacticaseibacillus rhamnosus*, *Ligilactobacillus hayakitensis* and *Limosilactobacillus vaginalis*) and *Bifidobacterium colobi*, and suppresses opportunistic pathogens including *Desulfovibrionaceae*, *Bacteroides* and *Helicobacteraceae*. By remodeling the intestinal microecology, these metabolites selectively promote the proliferation of specific probiotics while inhibiting the growth of opportunistic pathogens, thereby regulating the “intestinal-kidney axis” function and indirectly but precisely influencing systemic uric acid homeostasis.

In conclusion, the purified metabolites and extracts precisely identify and regulate specific targets, including XOD, URAT1, ABCG2, NF-κB, NLRP3, and TGF-β1, achieving precise intervention in the entire uric acid pathway, from “production - excretion - damage.” The multi-target, yet clearly defined, pharmacological characteristics of these interventions provide a unique molecular template and action paradigm for the development of a new generation of highly selective and low-side-effect uric acid-lowering drugs. As summarized in Tables 1, 2, purified metabolites and extracts exert therapeutic effects on hyperuricemia and its complications via multi-target actions.

**TABLE 1:** Purified metabolites in the treatment of hyperuricemia.

Metabolite	Source	Disease - model	Mechanisms	References
esculetin, esculin, fraxetin, fraxin	*Fraxinus chinensis* Roxb. (Oleaceae)	Potassium oxonate (i.g.)	↓ GLUT9, URAT1; ↑OAT1, OCT1, OCT2, OCTN1, OCTN2; anti-oxidation	Huang et al. (2023), Li et al. (2022)
Galangin	*Alpinia officinarum Hance* (Zingiberaceae)	NRK-52E cells	↓TNF-α, IL-1β,IL-18, PGE2,NO ↓NF-κB, PI3K/AKT; ↓NLRP3, ASC	Lu et al. (2019)
Total glucosides of Paeoniflora	*Paeonia lactiflora* Pall. (Paeoniaceae)	Adenine (i.g.)	↓ URAT1, GLUT9; ↑OAT1; ↓MCP-1, TNF-α; renal protection	Kang et al. (2020)
5-O-caffeoylshikimic acid (5OCSA)	*Smilax glabra* Roxb. (Smilacaceae)	Potassium oxonate (i.p.)	↓ XOD; ↓ TNF-α, IL-1β, IL-6, IL-18; renal protection	Zhang et al. (2021)
Berberine	*Phellodendron chinense* C.K.Schneid. (Rutaceae)	Potassium oxonate (i.p.)	↓ NLRP3 inflammasome; ↓URAT1	Chen et al. (2023), Li et al. (2021)
Polydatin	*Reynoutria japonica* Houtt. (Polygonaceae)	Fructose-fed	↓ XOD; ↓ NF-κB p65, COX-2, iNOS; ↓TNF-α, PGE2, IL-1β; renal protection	Chen et al. (2023), Wu et al. (2014)
Astilbin	*Smilax china* L. (Smilacaceae)	Potassium oxonate	↓ XOD; ↓ NF-κB p65, COX-2, iNOS; ↓TNF-α, PGE2, IL-1β; renal protection	Wang et al. (2016)
Tanshinone IIA	*Salvia miltiorrhiza* Bunge (Lamiaceae)	Adenine-fed	↓ MCP-1, IL-1β; ↓NF-κB nuclear translocation	Wu et al. (2012)
Gallic acid	*Sonneratia apetala* Banks (Lythraceae)	Potassium oxonate (i.p.)	↓ XOD; ↓ MDA, IL-6, IL-1β, TNF-α, TGF-β1; anti-oxidation	Jiang et al. (2022)
Emodin	*Rheum palmatum* L. (Polygonaceae)*,Reynoutria japonica* Houtt	Potassium oxonate (i.p.)	↑ fractional excretion of uric acid; ↓ IL-1β, IL-6, TNF-α	Hou et al. (2023)
Polygonati Rhizoma polysaccharide	*Polygonatum sibiricum Redouté* (Asparagaceae)	Potassium oxonate + hypoxanthine (i.g.)	↓ XO, ADA; ↑HGPRT; ↓URAT1; ↑ OAT1, OAT3; renal protection	Zhang et al. (2024)
Resveratrol	---	High-fat diet, FMT	↑ intestinal flora regulation; anti-inflammatory; renal protection	Shi et al. (2012), Zhou et al. (2024)
Hispidulin	*Plantago asiatica* L. (Plantaginaceae)	Adenine + potassium oxonate (i.g.)	↓ NLRP3 inflammasome, NF-κB; renal protection	Li et al. (2024)
Punicalagin	*Punica granatum* L. (Lythraceae)	Adenine + potassium oxonate (i.g.)	↓ URAT1, GLUT9; ↑ABCG2, OAT1; ↓IL-1β, IL-6, TNF-α; gut microbiota regulation; renal protection; glucose metabolism regulation	Han et al. (2025)

**TABLE 2:** Extracts in the treatment of hyperuricemia.

Extract	Extraction method	Disease model	Mechanisms	References
*Rhizoma smilacis glabrae* extract	Aqueous extract	Potassium oxonate (p.o.)	↓ hepatic XOD; ↓IL-10; renal and joint protection	Liang et al. (2019)
*Alpinia oxyphylla* Miq. (Zingiberaceae) seed extract	30% ethanol	Potassium oxonate (i.p.)	↓ URAT1; ↑ OAT1; ↓IL-1β, IL-6, TNF-α	Lee et al. (2019)
*Alpinia oxyphylla* Miq. (Zingiberaceae) seed extract combined with allopurinol	30% ethanol	Potassium oxonate (i.p.)	↓ serum and liver XOD; ↑ OAT1; ↓URAT1; ↓ IL-1β; renal protection	Sung and Kim (2022)
*Hovenia acerba* Lindl. (Rhamnaceae)water extract	Water extract	Potassium oxonate (i.p.) + hypoxanthine (i.g.)	↓ liver and serum XOD; ↑ ABCG2, OAT1; ↓ URAT1, GLUT9; regulation of injury-related genes; renal protection	Wang et al. (2025)
*Gymnadenia conopsea* (L.) R. Br. (Orchidaceae) ethanol extract	95%, 75% ethanol	Potassium oxonate + xanthine sodium salt	↓ XOD; regulation of metabolic pathways; anti-oxidation	Chen et al. (2022)
*Aconitum carmichaelii Debeaux* (Ranunculaceae) Cocta	Aqueous extract of Aconitum carmichaelii Debeaux	MSU-induced gouty arthritis	↓ caspase-1, SYK, PTGS2 mRNA; regulation of TNF signaling	Ye et al. (2021)
*Paeonia veitchii Lynch* (Paeoniaceae) extract	75% ethanol	Potassium oxonate (i.p.) + hypoxanthine (i.g.)	↓ XOD; ↓ URAT1, GLUT9; ↑ OAT1, ABCG2; hepatic and renal protection	Du et al. (2024)
*Dendrobium loddigesii* Rolfe (Orchidaceae)extract	80% ethanol	Potassium oxonate (i.p.)	Regulation of uric acid transporters; ↓TLRs/MyD88/NF-κB	Zhang et al. (2020)
*Dendropanax morbiferus* H.Lév. (Araliaceae) leaf ethanol extract	Water, 30%, 50%, 70% ethanol	Potassium oxonate (i.p.)	↓ XOD; renal protection; anti-oxidation	Lee et al. (2021)
*Liriodendron chinense* (Hemsl.) Sarg. (Magnoliaceae) bark ethanol extract	Ethanol	Adenine + potassium oxonate (i.g.)	↓ NF-κB, ASK1/JNK/c-Jun; ↓renal fibrosis; ↑OAT1, OAT3, ABCG2	Pan et al. (2021)
*Maclura cochinchinensis* (Lour.) Corner (Moraceae) heartwood extract	70% ethanol	Potassium oxonate (i.p.)	↓ XOD; ↓LPS-induced inflammatory genes; anti-oxidation	Sato et al. (2020)
Tu-Teng-Cao extract (*Psychotria serpens* L. (Rubiaceae))	Water extract	MSU-induced arthritis; hyperuricemia model	↓ NLRP3 inflammasome; ↓TNF-α, IL-6, IL-1β; ↓ MAPK/NF-κB; regulation of uric acid metabolism; ↓ kidney damage	Yao et al. (2020)
DKB114	Ethanol	Potassium oxonate (i.p.); clinical trial	↓ XOD; ↓ URAT1, GLUT9 protein	Lee et al. (2018a), Park et al. (2020)
Agrimonia pilosa Ledeb. (Rosaceae) and Salvia miltiorrhiza Bunge mixture	50% ethanol	Intra-articular injection	Anti-inflammatory (mechanism not fully confirmed)	Hwang et al. (2018)

### 6.2 Systemic effects of single botanical drugs

The various natural active metabolites in single botanical drugs, through their complex chemical properties, can provide interventions across multiple pathological and physiological processes involved in hyperuricemia. This intervention is characterized by multi-component and multi-targeted pharmacological effects. Compared to chemical synthetic drugs that target a single receptor or enzyme, these botanical drugs offer a broader range of therapeutic options and may reduce the risk of drug resistance associated with single-target therapies.

In the process of inhibiting uric acid production, multiple single botanical drugs exert inhibitory effects by targeting different nodes of the purine metabolism pathway. For example, *Piper longum* L. (Piperaceae), *Plantago asiatica* L (Plantaginaceae), and *Ilex cornuta* Lindl. and Paxton (Aquifoliaceae) all inhibit the activity of xanthine oxidase (XOD) in the liver. The active metabolite 6-Hydroxyluteolin in *P. asiatica* L. simultaneously inhibits the activities of both XOD and xanthine dehydrogenase (XDH). *Atractylodes macrocephala* Koidz. (Asteraceae) downregulates the levels of two key enzymes, adenosine deaminase and XOD, in the liver. Additionally, *Cichorium glandulosum* Boiss. and A. Huet (Asteraceae) reduces the absorption of exogenous purines by inhibiting the intestinal purine transporter CNT2, thereby decreasing the precursor substances for uric acid synthesis at the source.

In promoting uric acid excretion, the regulatory effects of single botanical drugs are often characterized by bidirectional, multi-target modulation of the uric acid transporter network in both the kidneys and the intestines. This regulatory mechanism extends beyond a single transporter, simultaneously influencing both the reabsorption and excretion processes of uric acid. For example, *Orthosiphon aristatus* (Blume) Miq. (Lamiaceae) significantly increases the expression of ABCG2 in the intestine, promoting uric acid excretion, and decreases the expression of GLUT9. *Euodia rutaecarpa* (Juss.) Benth (Rutaceae) downregulates the expression of URAT1 and GLUT9, while upregulating the expression of OAT1 and ABCG2, thereby forming a comprehensive effect that enhances the net excretion of uric acid. This coordinated regulation of the “kidney-intestine” dual-channel excretory system presents potential therapeutic advantages, especially for patients with renal dysfunction.

In terms of anti-inflammatory and kidney protection, single botanical drugs exert their effects by regulating multiple key inflammatory and fibrosis signaling pathways. The mechanisms include not only the inhibition of NLRP3 inflammasome activation but also extensive modulation of core pathways such as NF-κB, PI3K/AKT, and TGF-β/Smad. For instance, *A. macrocephala* can reduce the levels of inflammatory factors such as IL-1β and TNF-α and regulate macrophage polarization. *Eucommia ulmoides* Oliv. (Eucommiaceae) leaves reduce inflammatory damage by downregulating the expression of Toll-like receptor 4 (TLR4) in the kidneys. *Reynoutria japonica* Houtt. (Polygonaceae) (formerly *Polygonum cuspidatum*) modulates multiple inflammatory-related signaling molecules, including TLR4, NLRP3, and monocyte chemoattractant protein-1 (MCP-1). *Ampelopsis grossedentata* (Hand.-Mazz.) W.T.Wang (Vitaceae) alleviates oxidative damage by reducing malondialdehyde levels and increasing superoxide dismutase (SOD) activity. Regarding anti-renal fibrosis, *Sophora flavescens* Aiton (Fabaceae) exerts protective effects by regulating the TGF-β1/Smad and NF-κB signaling pathways. *Orthosiphon aristatus* (Blume) Miq. Inhibits NF-κB phosphorylation, downregulates the expression of α-smooth muscle actin (α-SMA) and vimentin, and upregulates the expression of E-cadherin, thereby inhibiting epithelial-mesenchymal transition (EMT) and improving renal fibrosis.

Certain single botanical drugs also affect urate metabolism by regulating gut microbiota and renal function. For example, *Terminalia chebula* Retz. (Combretaceae) increases beneficial bacteria and reduces harmful bacteria by upregulating the expression of tight junction proteins (ZO-1, Occludin, and Claudin-1), which in turn enhances intestinal barrier function. It also reduces levels of D-lactate (D-Lac), diamine oxidase (Marques de Mattos et al., 2012), and lipopolysaccharide (LPS) in the serum. *Orthosiphon aristatus* (Blume) Miq (commonly known as kidney tea) increases the abundance of beneficial bacteria such as *Dubosilla* and *Bifidobacterium*, modulating intestinal bacterial metabolites, improving gut microbiota structure, and influencing urate metabolism and excretion through related metabolic pathways.

Several single botanical drugs lower uric acid levels partly by modulating specific gut microbial communities. For example, Terminalia chebula Retz. Enriches beneficial genera such as *Roseburia*, *Monoglobus* and *DNF00809*, while suppressing opportunistic pathogens including *Clostridium_innocuum_group*, *Erysipelatoclostridium*, *UBA 1819*, *Subdoligranulum* and *Clostridium_sensu_stricto_1*. Similarly, Orthosiphon aristatus (Blume) Miq (kidney tea) increases the abundance of *Roseburia* and *Enterorhabdus*, and decreases *Ileibacterium* and *UBA 1819*. These shifts in gut microbiota composition are associated with reduced serum urate levels and improved metabolic outcomes, suggesting that targeted regulation of intestinal bacteria constitutes a previously underappreciated mechanism underlying the anti-hyperuricemic action of certain single botanical drugs.

As summarized in Table 3, single botanical drugs exert therapeutic effects on hyperuricemia and its complications via multi-target actions. However, the available evidence largely derives from preclinical models, and further rigorous clinical studies are required to validate these effects in humans. In conclusion, the multi-target effects of these single botanical drugs provide a promising therapeutic strategy for managing hyperuricemia and its complications, while also offering potential benefits for the prevention and treatment of related metabolic disorders.

**TABLE 3:** Single botanical drugs in the treatment of hyperuricemia.

Single botanical drugs	Classification of drug efficacy	Disease - model	Mechanisms	References
*Cordyceps militaris* (L.) Fr. (Cordycipitaceae)	Yang tonic	Potassium oxonate (i.p.) + hypoxanthine (p.o.)	↓ URAT1	Jiang et al. (2024)
*Atractylodes macrocephala* Koidz. (Asteraceae)	Tonifying medicine	Potassium oxonate (i.p.) + yeast powder (i.g.)	↓ XOD, ADA; ↓IL-1β, TNF-α, NF-κB; ↓ macrophage M1 polarization; renal protection	Qian et al. (2023)
*Piper longum* L. (Piperaceae)	Warm-inside medicine	Hypoxanthine (i.g.) + potassium oxonate (i.p.)	↓ MAPK/PI3K-AKT; anti-inflammatory; ↓XOD; ↑ OAT1, OAT3; ↓ URAT1, GLUT9	Wu et al. (2024)
*Isaria cicadae* Miq. (syn. Cordyceps chanhua)	Yang tonic	Acute: hypoxanthine + potassium oxonate; Chronic: adenine + ethambutol (i.p.)	↓ XOD; ↓ NF-κB; anti-inflammatory; renal protection	Dong et al. (2023)
*Plantago asiatica* L. (Plantaginaceae)	Moisturizing drug	Potassium oxonate (i.g.)	↓ XOD; ↓ URAT1, GLUT9; ↑ PPAR; regulation of lipid and amino acid metabolism	Liu et al. (2024)
*Eucommia ulmoides* Oliv. (Eucommiaceae) leaves	Yang tonic	High-fat + high-fructose diet	↓ inflammatory factors; ↓ NF-κB; ↓GLUT9; renal protection	Fang et al. (2019), Gong et al. (2022)
*Tetragonia tetragonoides* (Pall.) Kuntze (Aizoaceae)	Moisturizing medicine	High-fat diet	↓ XOD; regulation of lipid metabolism	Lee et al. (2018b)
*Ilex cornuta* Lindl. and Paxton (Aquifoliaceae)	Heat-clearing medicine	Potassium oxonate (p.o.) + high-purine diet	↓ XOD; ↑ ABCG2; ↓GLUT9, URAT1; improved renal function; anti-oxidation	Mao et al. (2025)
*Terminalia chebula* Retz. (Combretaceae)	Astringent drug	High-fructose solution + potassium oxonate (i.g.)	↓ XOD; gut microbiota regulation; ↑ intestinal barrier; ↓IL-6, TNF-α	Liu et al. (2024)
*Plumeria rubra* L. (Apocynaceae)	Antipyretic	Potassium oxonate (i.p.)	↓ XOD; anti-oxidation	Mohamed Isa et al. (2018)
*Reynoutria japonica* Houtt. (Polygonaceae)	Heat and dampness medicine	Adenine (i.g.) + yeast feed	↑ AMPK, FOXO3α; ↓TLR4, NLRP3, MCP-1	Ma et al. (2019)
*Astragalus mongholicus* Bunge (Fabaceae)	Qi-tonifying medicine	Adenine + potassium oxonate (i.g.)	↑ ABCG2; ↓ URAT1, GLUT9; ↓ XOD; renal protection	Zhang et al. (2023)
*Fraxinus chinensis Roxb.* (Oleaceae)	Heat-clearing and dampness-drying medicine	Potassium oxonate (p.o.)	↓ URAT1, GLUT9; ↑OAT1, OCT1; renal protection	Wang et al. (2017)
*Camellia japonica* L	Heat suppressant	Potassium oxonate (i.p.)	↓ XOD; anti-oxidation; lipid metabolism regulation; anti-inflammatory	Yoon et al. (2017)
*Orthosiphon aristatus* (Blume) Miq. (Lamiaceae)	Moisturizing herb	Potassium oxonate (i.g.)/high-fructose diet	↓ XOD; ↑ intestinal ABCG2; ↓ intestinal GLUT9; ↓ EMT; regulation of lipid/amino acid metabolism; gut flora regulation	Wu et al. (2023)
*Euodia rutaecarpa* (Juss.) Benth. (Rutaceae)	Antipyretic agent	Potassium oxonate (p.o.) + high-fat diet	↓ NLRP3 inflammasome; ↓URAT1, GLUT9; ↑OAT1, ABCG2	Wang et al. (2024)
*Ampelopsis grossedentata* (Hand.-Mazz.) W.T.Wang	Heat and blood cooler	Adenine + potassium oxonate (i.g.)	↓ XOD; ↓ GLUT9, URAT1; anti-inflammatory; anti-oxidation; renal protection	Zhou et al. (2024)
*Cichorium glandulosum* Boiss. and A. Huet	Wind-dampness medicine	Potassium oxonate + adenine (i.g.)	↓ uric acid; ↓ PTGS2 (inflammation); lipid metabolism regulationregulation of lipid metabolism	Yang et al. (2024)

### 6.3 Effect of TCM formulas

Under the guidance of the holistic concept and the theory of syndrome differentiation and treatment in Traditional Chinese Medicine, TCM formulas, through the organic combination of *sovereign, minister, assistant, and messenger botanical drugs*, are intended to produce a network of multi-component, multi-target effects. This approach demonstrates systematic therapeutic advantages for hyperuricemia and its complications, such as gouty arthritis and uric acid nephropathy. The core mechanism can be summarized in four interrelated levels: “reducing generation at the source, promoting excretion through pathways, overall regulation of metabolism, and targeted organ protection,” which together constitute an integrated intervention strategy.

#### 6.3.1 Multi-pathway regulation of urate metabolism, effectively lowering serum urate levels

The regulation of urate metabolism by TCM formulas is bidirectional and multi-channel. On one hand, they reduce uric acid synthesis by inhibiting the activity of key enzymes such as xanthine oxidase (XOD) and adenosine deaminase in the liver. For instance, Simiao Decoction, Guizhi Shaoyao Zhimu Decoction (GSZD), and TongFengXiaoFang (TFXF) all demonstrate clear inhibitory effects on uric acid-generating enzymes. On the other hand, these formulas significantly enhance uric acid excretion by precisely regulating the expression of uric acid transport proteins in the kidneys and intestines. In the kidneys, formulas such as Biqi Capsules (BQ) and Fangji Huangqi Decoction (FHT) can downregulate the expression of reabsorption proteins GLUT9 and URAT1, while upregulating the expression of excretion proteins OAT1, OAT3, and ABCG2, thereby enhancing the kidneys’ ability to excrete uric acid.

In the intestines, formulas such as Qu-zhuo-tong-bi Decoction (QZTBD) and Dendrobium Officinalis Six Nostrum (DOS) upregulate the expression of intestinal excretion protein ABCG2 and modulate the intestinal microbiota with specific bacterial taxa. QZTBD increases the abundance of *Bacteroidetes* and butyrate-producing genera such as *Butyricicoccus*, elevates the levels of short-chain fatty acids (acetate, propionate and butyrate), and upregulates the expression of tight junction proteins (ZO-1, Occludin), thereby enhancing intestinal barrier function and promoting uric acid decomposition and excretion. Similarly, DOS modulates intestinal microecology by enriching beneficial bacteria like *Akkermansia* and *Lactobacillus*. Other TCM formulas further support this mechanism: for instance, Bining Decoction increases *Turicibacter* and adjusts the Firmicutes/Bacteroidetes ratio, while Guizhi Shaoyao Zhimu Decoction elevates *Lactobacillus*, *Ruminococcaceae* and *Turicibacter*, and reduces pro-inflammatory *Blautia*. These coordinated actions on the gut microbiota, together with enhanced ABCG2-mediated uric acid excretion, constitute a novel paradigm of “renal-intestinal coordinated excretion”.

#### 6.3.2 Multi-target inhibition of the inflammatory response, improving the metabolic disorder state

Hyperuricemia is often accompanied by chronic low-grade inflammation and metabolic syndrome. TCM formulas exert powerful anti-inflammatory and metabolic regulatory effects by interfering with multiple key signaling pathways. In terms of anti-inflammatory effects, many formulas, such as Gegen Qinlian Decoction (GGQLD) and Simiao Pill Module (SMM), can inhibit the activation of the NF-κB pathway, blocking the assembly and activation of the NLRP3 inflammasome, and effectively reducing the maturation and release of core inflammatory factors like IL-1β and IL-18. Furthermore, different formulas have different focuses: for example, Dispelling Dampness, Relieving Turbidity, and Dredging Collaterals Decoction (DED) targets the inhibition of the IL-17 signaling pathway, Bining Decoction (BN) regulates the JAK/STAT pathway, and Shenling Baizhu San (SLBZ) reduces inflammation through the CDK5/PPARγ pathway. These effects together alleviate inflammatory damage in tissues such as joint synovium and kidneys. In terms of metabolic regulation, these formulas not only limit purine metabolism but also improve lipid and amino acid metabolism. For example, TongFengXiaoFang (TFXF) and Er-Miao-Wan Formula (EMW) can correct multiple metabolic disorders induced by high uric acid, creating a favorable internal environment for disease treatment.

Additionally, many TCM formulas, such as Bining Decoction (BN) and Guizhi Shaoyao Zhimu Decoction (GSZD), can also reshape the intestinal microecology. Moreover, based on current evidence, the aforementioned effects of traditional Chinese medicine may be closely associated with the regulation of the gut–kidney axis. By acting on the intestinal-kidney axis, they not only reduce the intestinal source of endogenous uric acid but also produce beneficial metabolites (such as short-chain fatty acids) that enhance the intestinal barrier, reduce systemic low-grade inflammation caused by endotoxin translocation, and indirectly affect the inflammatory state and function of the kidneys through the circulatory system. This exerts a comprehensive effect on urate metabolism and target organ function.

#### 6.3.3 Multiple mechanisms to protect target organs, delaying the progression of complications

Protecting the kidneys and preventing structural damage to joints are critical therapeutic goals. TCM formulas reduce damage to target organs caused by hyperuricemia through multiple mechanisms. Regarding kidney protection, these formulas demonstrate various functions: first, anti-apoptotic effects, such as Renyao Formula, which regulates the Bcl-2/Bax ratio, and Shizhifang Decoction (SZF), which inhibits the ERK1/2/caspase-3 pathway, protecting renal tubular epithelial cells; second, anti-fibrotic effects, such as Biqi Capsules (BQ) and Qizhu Xie Zhuo Fang (QZXZF), which inhibit epithelial-mesenchymal transition (EMT), and Cichorium intybus L. Formula (CILF), which downregulates the expression of fibrosis-related genes like STAT3 and VEGFA, effectively delaying renal interstitial fibrosis; third, direct reduction of pathological damage, such as Fufang Qiling Granules (FQG), which reduce the deposition of urate crystals in the kidneys and the infiltration of interstitial inflammation.

In terms of joint protection, formulas like Sanmiao Wan (SMW) reduce osteoclast generation by regulating the balance of Th17 cells and inhibiting the RANKL/RANK pathway, thereby preventing osteoporotic bone erosion caused by gout.

#### 6.3.4 Advantages of overall regulation and individualized treatment

It is important to note that all these mechanisms often operate together within the same formula. For example, Simiao Pill Module (SMM) not only inhibits XOD to reduce uric acid levels but also exhibits anti-inflammatory and renal protective effects, demonstrating the overall regulatory feature of TCM formulas in “treating both the symptoms and the root cause.” Additionally, based on TCM syndrome differentiation, different formulas focus on specific therapeutic methods. For instance, the methods of clearing heat and removing dampness, tonifying the spleen and clearing turbidity, and activating blood circulation correspond to different formulas, achieving individualized precise intervention within the framework of overall regulation.

**FIGURE 3:**
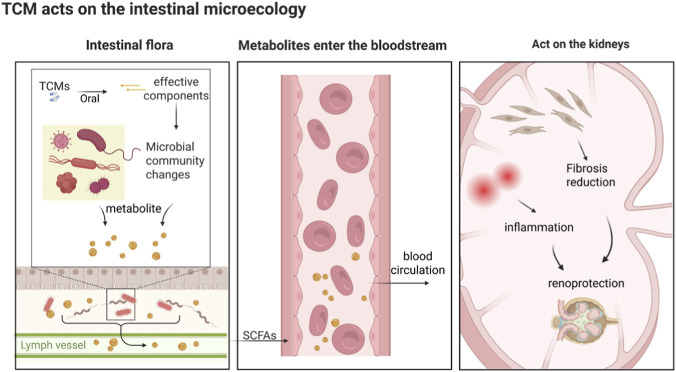
Modulation of intestinal microecology by TCM. Traditional Chinese medicine (TCM) reshapes gut microbial composition and metabolites, which subsequently enter the circulation and exert renoprotective effects by alleviating inflammation and fibrosis in the kidneys. Created in BioRender. LI, H. (2026) https://BioRender.com/2ubqv7.

In conclusion, the treatment of hyperuricemia with TCM formulas is not a single-stage blockade but forms a three-dimensional and integrated treatment system through the ingenious combination of “king, minister, assistant, and messenger.” It starts by reducing the core indicator of serum urate, regulates the systemic inflammatory and metabolic networks, and precisely protects the kidneys and joints. This multi-dimensional and systematic intervention model is a concentrated reflection of the unique advantages and modern scientific connotations of TCM formulas in treating complex diseases. As summarized in Table 4, TCM formulas exert therapeutic effects on hyperuricemia and its complications via multi-target actions. The TCM acts on the intestinal microecology are shown in Figure 3.

**TABLE 4:** TCM formulas in the treatment of hyperuricemia.

TCM formulas	Ingredients	Disease/syndrome type	Mechanisms	References
Bining Decoction	*Atractylodes lancea* (Thunb.) DC. (Asteraceae), *Dioscorea hypoglauca* Palib. (Dioscoreaceae), *Plantago asiatica* L. (Plantaginaceae)	Cold-damp obstruction syndrome	↑ intestinal flora; ↑uric acid decomposition; ↓intestinal purine absorption; ↓JAK/STAT	Huang et al. (2022)
Biqi Capsules	*Strychnos nux-vomica* L. (Loganiaceae), *Codonopsis pilosula* (Franch.) Nannf. (Campanulaceae), *Atractylodes macrocephala* Koidz. (Asteraceae)	Cold-damp obstruction syndrome	↓ GLUT9; ↑ OAT1; ↓TLR4/NLRP3; ↓ADA; ↓ EMT; anti-inflammatory	Li et al. (2025)
Shenling Baizhu San	*Panax ginseng* C.A.Mey. (Araliaceae), *Poria cocos* (Schwein.) F.A.Wolf (Fomitopsidaceae), *Atractylodes macrocephala* Koidz. (Asteraceae)	Spleen deficiency and dampness excess syndrome	↑ PPARγ phosphorylation; ↓uric acid	Wang et al. (2022)
Er-Miao-Wan Formula	*Phellodendron chinense* C.K.Schneid. (Rutaceae), *Atractylodes lancea* (Thunb.) DC. (Asteraceae)	Damp-heat obstruction syndrome	Regulation of purine, lipid, amino acid metabolism; ↑uric acid excretion	Gu et al. 2023; Huang et al., 2019)
Fangji Huangqi Decoction	*Stephania tetrandra* S.Moore (Menispermaceae), *Astragalus mongholicus* Bunge (Fabaceae), *Atractylodes macrocephala* Koidz. (Asteraceae), *Glycyrrhiza uralensis* Fisch. ex DC. (Fabaceae)	Spleen deficiency and dampness excess syndrome	↑ OAT1, OAT3, ABCG2; ↓ IL-1β; ↓NF-κB; renal protection	Xing et al. (2020)
Fufang Qiling Granules	*Astragalus mongholicus* Bunge (Fabaceae), *Smilax glabra* Roxb. (Smilacaceae), *Taxillus chinensis* (DC.) Danser (Loranthaceae)	Dampness-heat trapping the spleen syndrome	↓ XOD; ↑ renal function; ↑ uric acid excretion	Ye et al. (2024)
Compound Tufuling Oral-Liquid	*Smilax glabra* Roxb. (Smilacaceae), *Dioscorea septemloba* Thunb. (Dioscoreaceae) [or *Dioscorea spongiosa* J.Q.Xi, M.Mizuno and W.L.Zhao], *Curcuma longa* L. (Zingiberaceae)	Dampness-heat poison accumulation syndrome	↓ XOD-producing bacteria; ↓ uric acid production; gut microbiota regulation; ↑intestinal microecology	Gao et al. (2020)
Modified Baihu Decoction	Gypsum, *Anemarrhena asphodeloides* Bunge (Asparagaceae), *Paeonia lactiflora* Pall. (Paeoniaceae), *Achyranthes bidentata* Blume (Amaranthaceae)	Qi-level heat excess syndrome	Gut microbiota structure regulation; ↓ uric acid; ↓ IL-1β, TGF-β1	Wang et al. (2022)
Gegen Qinlian Decoction	*Pueraria montana* var. *Lobata* (Willd.) Maesen and S.M.Almeida ex Sanjappa and Predeep (Fabaceae), *Scutellaria baicalensis* Georgi (Lamiaceae), *Glycyrrhiza uralensis* Fisch. ex DC. (Fabaceae), *Coptis chinensis* Franch. (Ranunculaceae)	Dampness-heat trapping the spleen syndrome	↓ NLRP3 inflammasome; ↓URAT1, GLUT9; ↓ apoptosis; renal protection	Wang et al. (2021)
Guizhi Shaoyao Zhimu Decoction	*Cinnamomum cassia* (L.) J.Presl (Lauraceae), *Cynanchum otophyllum* C.K.Schneid. (Apocynaceae), *Anemarrhena asphodeloides* Bunge (Asparagaceae)	Cold-damp obstruction syndrome	↓ ADA; regulation of metabolic pathways; ↑intestinal flora; ↑intestinal barrier; ↓ TLR4, MYD88, p-NF-κB p65	Bian et al. (2024)
Cichorium intybus L. Formula	*Cichorium intybus* L. (Asteraceae), *Gardenia jasminoides* J.Ellis (Rubiaceae), *Pueraria montana* var. *Lobata* (Willd.) Maesen and S.M.Almeida ex Sanjappa and Predeep (Fabaceae)	Damp-heat obstruction syndrome	↓ IL-17, TNF, AGE-RAGE; anti-inflammatory; renal protection; delay fibrosis	Amatjan et al. (2023)
Dispelling Dampness, Relieving Turbidity and Dredging Collaterals Decoction	*Dioscorea* spp. (Dioscoreaceae), *Smilax glabra* Roxb. (Smilacaceae), *Phellodendron chinense* C.K.Schneid. (Rutaceae)	Phlegm-dampness and blood stasis syndrome	↓ XOD; ↓AGE-RAGE, IL-17; ↓ fibrosis; renal protection	Liu et al. (2023)
Renal Herb Formula	*Astragalus mongholicus* Bunge (Fabaceae), *Lonicera japonica* Thunb. (Caprifoliaceae), *Imperata cylindrica* (L.) P.Beauv. (Poaceae), *Plantago asiatica* L. (Plantaginaceae)	Liver and kidney deficiency syndrome	↓ uric acid; ↓apoptosis; ↓NF-κB-related inflammation; ↓p-p65, c-Cas-1, IL-1β, IL-18	Tang et al. (2023)
Shizhifang Decoction	*Plantago asiatica* L. (Plantaginaceae), *Sinapis alba* L. (Brassicaceae), *Vaccaria hispanica* (Mill.) Rauschert (Caryophyllaceae), *Malva verticillata* L. (Malvaceae)	Excessive heat and toxin syndrome	↓ uric acid; ↓AGE-RAGE, PI3K-Akt, IL-17; ↓inflammation; renal protection	Wu et al. (2024)
Simiao Decoction	*Phellodendron chinense* C.K.Schneid. (Rutaceae), *Atractylodes lancea* (Thunb.) DC. (Asteraceae), *Achyranthes bidentata* Blume (Amaranthaceae), *Coix lacryma-jobi* var. *Ma-yuen* (Rom.Caill.) Stapf (Poaceae)	Damp-heat obstruction syndrome	↓ XOD, ADA; ↓IL-1β, IL-9, IFN-γ, MIP-1α, MIP-1β; ↑ABCG2, OAT1; ↓ GLUT9, URAT1, OAT4; lipid metabolism regulation; renal protection; EMT inhibition; gut microbiota regulation	Lin et al. (2020), Shui et al. (2022), Zeng et al. (2024)
Dendrobium Officinalis Six Nostrum	*Dendrobium officinale* Kimura and Migo (Orchidaceae), *Phellodendron chinense* C.K.Schneid. (Rutaceae), *Atractylodes lancea* (Thunb.) DC. (Asteraceae)	Liver and kidney deficiency syndrome	↑ ABCG2, PDZK1; ↓ GLUT9; ↓ XOD; ↑ intestinal barrier; lipid metabolism regulation; ↓NLRP3, Caspase-1, TLR4	Ge et al. (2023), Guo et al. (2020)
Tong Feng Kang	*Smilax glabra* Roxb. (Smilacaceae), *Euonymus alatus* (Thunb.) Siebold (Celastraceae), *Coix lacryma-jobi* var. *Ma-yuen* (Rom.Caill.) Stapf (Poaceae)	Damp-heat and blood stasis syndrome	↓ uric acid; ↓IFN-γ, IL-1β, IL-2, IL-4, IL-13, TNF-α, MCP-1; renal protection	Wang et al. (2024)
Tongfengxiaofang	*Viola philippica* Cav. (Violaceae), *Taraxacum mongolicum* Hand.-Mazz. (Asteraceae), *Lysimachia christinae* Hance (Primulaceae)	Damp-heat obstruction syndrome	↓ XOD; ↓ URAT1, GLUT9; ↑ OAT1; regulation of metabolic pathways	Chen et al. (2021)
DaiTongXiao	*Elsholtzia rugulosa* Hemsl. (Lamiaceae), *Pinus tabuliformis* Carrière (Pinaceae)	Dampness-heat poison accumulation syndrome	↓ uric acid; ↓TLR4/MyD88/NF-κB; anti-inflammatory; ↓ renal fibrosis; renal protection	Liu et al. (2024)

## 7 Discussion and prospect

Purified metabolites, extracts, single botanical drugs, and TCM formulas each have their distinct characteristics and multi-target mechanisms in reducing uric acid levels. Purified metabolites exert precise actions, targeting key processes such as production, excretion, and inflammation associated with uric acid. Single botanical drugs can influence urate metabolism from multiple aspects, while formulas harness the effects of multiple components and targets to achieve comprehensive metabolic regulation and symptom improvement. Together, these three elements form a complex and effective mechanistic network in TCM for lowering uric acid, offering a wider range of treatment options and promising research prospects for hyperuricemia management. Importantly, these four levels are not isolated categories but a progressive hierarchy: purified metabolites validate specific molecular targets (mechanistic probes), extracts and single botanical drugs achieve multi-component effects, and TCM formulas enable network regulation via the sovereign-minister-assistant-messenger principle. This integrative framework distinguishes our review by deconstructing formula-level efficacy into testable, evidence-based lead combinations.

A key feature of TCM in treating HUA is its multi-component nature, which allows for potential multi-target interactions. Unlike single-agent Western drugs that block only uric acid production or reabsorption, TCM formulas simultaneously intervene at multiple nodes: reducing synthesis via XOD/ADA inhibition, promoting renal excretion through URAT1/GLUT9 downregulation and ABCG2/OATs upregulation, enhancing intestinal excretion via ABCG2 and microbiota remodeling, and suppressing NLRP3/NF-κB-driven inflammation and oxidative stress. This multi-target integration may not only lower serum urate levels but also offer potential benefits against gouty arthritis, renal fibrosis, and metabolic disturbances—addressing the systemic nature of hyperuricemia. Compared with previous reviews that often examined these mechanisms separately, the present work underscores their coordinated, network-based interplay, which is the hallmark of TCM’s holistic philosophy.

### 7.1 Methodological considerations of preclinical studies

The multi-target framework described above is supported by a large body of preclinical studies. However, several methodological limitations should be considered when interpreting the findings. One concern is that most animal experiments rely on acute hyperuricemia models induced by potassium oxonate or intraperitoneal injection of uric acid precursors. These models produce transient urate spikes that do not recapitulate the chronic nature of human hyperuricemia. Moreover, many studies administer test botanical drugs prophylactically rather than treating established hyperuricemia, which creates a gap between experimental design and clinical reality.

The reliance on acute, chemically-induced models has profound implications for data interpretation. Potassium oxonate is a uricase inhibitor, a mechanism not relevant to human physiology where uricase is already non-functional. A botanical drug showing potent XOD inhibition in an acute oxonate model may have limited efficacy in a setting of chronic, transporter-driven hyperuricemia. Furthermore, the prophylactic administration design (giving the TCM intervention before or simultaneously with the uricase inhibitor) tests a preventive effect, whereas clinical use would be therapeutic. This discrepancy likely overestimates effect sizes. Consequently, preclinical findings from such models should be considered hypothesis-generating. To improve translational value, future studies should use chronic models (e.g., genetically modified uricase-knockout mice, or long-term high-fructose/purine diets) and adopt a treatment-only design after hyperuricemia is firmly established. Only then can the data more reliably predict clinical outcomes.

Another issue relates to dosing practices. Many studies use intraperitoneal injection even for metabolites with known low oral bioavailability (e.g., quercetin, berberine), generating efficacy data that may not translate to oral human use. Positive controls such as allopurinol are frequently used at supratherapeutic doses, which may unfairly advantage the test botanical drug. Additionally, crude extracts are often used without specifying marker plant metabolite concentrations or batch-to-batch variability, limiting reproducibility. Finally, negative results are rarely published, which may create an inflated perception of efficacy.

Taken together, the current evidence base for TCM in hyperuricemia should be regarded as hypothesis-generating rather than conclusive. Rigorous confirmation under conditions that better mimic human disease is needed.

### 7.2 Heterogeneity and unresolved questions in mechanistic studies

Even among positive studies, variations and uncertainties exist. For example, URAT1 expression is reported to be downregulated by some botanical drugs (e.g., *Smilax glabra*, *Clerodendranthus spicatus*) but upregulated by others under similar experimental conditions. Whether these differences reflect genuine pharmacological variation, differences in disease models (acute vs. chronic), or assay artifacts (PCR vs. Western blot, different antibodies) is unclear. The relative contribution of each transporter to the overall urate-lowering effect is rarely quantified, and most studies focus on URAT1 while neglecting other equally important transporters such as OAT4, NPT1, and MRP4.

A critical gap pervading the current literature is that most mechanistic findings are correlational, not causal. A large number of studies report changes in gene/protein expression (e.g., downregulation of URAT1 or NLRP3 mRNA) or shifts in gut microbiota composition (e.g., increased *Lactobacillus*). However, these observations are merely associations. Several factors contribute to this stagnation. First, functional validation is often absent: demonstrating that a TCM extract reduces URAT1 protein does not prove that this reduction is responsible for the urate-lowering effect. Second, causality is rarely tested using rigorous methods. For the gut microbiota, for instance, few studies use fecal microbiota transplantation (FMT) from TCM-treated donors into germ-free recipients to prove that microbiota changes are sufficient to lower urate. Similarly, causal inference for specific transporters can be strengthened using conditional knockout animals or siRNA-mediated knockdown in target tissues (e.g., renal tubules), which are almost never performed. Third, multi-omics data are often reported descriptively without employing causal inference algorithms (e.g., Mendelian randomization, mediation analysis) to distinguish drivers from passengers.

To move the field forward, future research must prioritize functional validation. This includes: (1) transporter-specific knockdown/knockout in relevant cell lines or animal models; (2) FMT studies combined with gnotobiotic mice; and (3) application of statistical methods for causal inference on multi-omics datasets. Without such evidence, the field will remain at a descriptive, correlative stage, limiting its translational impact.

Furthermore, “multi-target” claims are often based on transcript or protein changes without functional validation. For instance, downregulation of NLRP3 mRNA does not necessarily translate into reduced IL-1β secretion; upregulation of ABCG2 protein does not always mean increased urate transport activity. Mechanistic studies that stop at expression analysis provide only correlative evidence.

In the gut microbiota domain, most TCM studies report descriptive shifts in microbial composition (e.g., increased *Lactobacillus*, decreased *Escherichia-Shigella*) but do not establish causality. Germ-free mouse experiments or fecal microbiota transplantation (FMT) from TCM-treated donors are rarely performed. Moreover, 16 S rRNA sequencing pipelines vary widely across studies, making cross-study comparisons difficult. Direct causal evidence linking TCM-induced microbiota changes to specific molecular pathways regulating uric acid transport remains lacking.

### 7.3 Gut microbiota and the gut–kidney axis: Current evidence

As noted above, although associative evidence links gut dysbiosis with hyperuricemia, and TCM can reshape microbial communities, several questions remain to be addressed. First, whether microbiota changes are a cause or a consequence of hyperuricemia remains debated. Second, studies that report correlations between specific bacterial taxa and serum urate levels rarely control for important confounders such as diet, medication use (including allopurinol), and comorbidities. Third, the proposed SCFAs–GPR43 axis has been demonstrated in other metabolic diseases but not systematically tested for urate handling. Therefore, while the gut–kidney axis is a conceptually attractive target, current evidence for its therapeutic modulation by TCM is preliminary and associative rather than causal.

### 7.4 Translational barriers

Several fundamental translational challenges must be explicitly acknowledged. The low oral bioavailability of many purified metabolites (e.g., quercetin <5%, berberine <2%) limits their therapeutic translation (Y. Guo and Bruno, 2015; Murakami, Bodor and Bodor, 2023). Yet most preclinical studies administer these metabolites intraperitoneally, generating efficacy data that are largely irrelevant to oral human use. Pharmacokinetic studies and novel drug delivery systems (e.g., nanoparticles, liposomes, co-crystallization) are urgently needed before clinical development can be seriously considered.

Safety assessment is another critically neglected dimension. Few studies report systematic evaluation of renal or hepatic toxicity beyond crude observation (e.g., body weight, general behavior). Histopathology of liver and kidney is occasionally performed but rarely quantified by blinded pathologists. Long-term safety studies (≥3 months) are almost nonexistent. Herb–drug interactions with commonly co-administered drugs (allopurinol, febuxostat, thiazide diuretics, ACE inhibitors) have not been systematically investigated. The historical example of aristolochic acid nephropathy serves as a stark reminder that “natural” does not equal “safe.”

Standardization of extracts and development of consistent quality control protocols remain primary barriers to clinical adoption. Overcoming these standardization gaps is essential for clinical translation. While the lack of batch-to-batch consistency currently limits reproducibility, this challenge also points to a clear priority for future research: the development of validated reference standards and quality control protocols that can support the evidence-based adoption of TCM.

### 7.5 Safety profile of anti-hyperuricemia TCM

A critical omission in many TCM studies, including those cited in this review, is the systematic evaluation of safety. The prevailing notion that “natural” equates to “safe” is misleading and potentially dangerous. While conventional urate-lowering drugs have well-documented adverse effects, TCM interventions are not devoid of risk.

Potential organ toxicity: Several botanical drugs commonly used for HUA contain plant metabolites with known nephrotoxic or hepatotoxic potential. For example, *Aristolochia species* (Aristolochiaceae) (though not a primary botanical drug for HUA) are a classic cause of aristolochic acid nephropathy. More relevant to this review, *E. rutaecarpa* contains evodiamine and rutaecarpine, which have been reported to cause hepatotoxicity at high doses (Nie et al., 2025; Yang et al., 2021). *Reynoutria japonica* (formerly *P. cuspidatum*) is rich in emodin and resveratrol, but emodin has potential nephrotoxic effects (Dong et al., 2016). Long-term use of *Phellodendron chinense* (containing berberine) may affect liver function (Wang et al., 2020). However, nearly all studies cited in Tables 1–4 either do not report safety data or only mention crude observations like body weight and general behavior. Kidney histopathology, when performed, is rarely quantified by blinded pathologists, and serum biomarkers of liver injury (ALT, AST) are inconsistently reported.

Herb-drug interactions: Patients with HUA often have comorbidities (hypertension, dyslipidemia, CKD) and take multiple medications (allopurinol, febuxostat, losartan, statins, SGLT2 inhibitors). TCM formulas, which contain dozens of metabolites, have a high potential for pharmacokinetic or pharmacodynamic interactions. For instance, many botanical drugs (e.g., *Glycyrrhiza uralensis,* Fisch. ex DC. (Fabaceae), licorice) can inhibit CYP450 enzymes or affect drug transporters (e.g., P-glycoprotein), potentially altering the levels of cAo-administered drugs (Chen et al., 2023). These interactions have not been systematically investigated.

Recommendations for safety assessment: Future preclinical and clinical studies must incorporate rigorous safety endpoints as primary outcomes, not afterthoughts. These include: (1) systematic histopathological examination of the liver and kidney by blinded pathologists; (2) routine measurement of liver enzymes, serum creatinine, and electrolytes; (3) long-term toxicity studies (≥3 months) in relevant animal models; (4) *in vitro* screening for CYP450 inhibition and transporter-mediated drug interactions. The TCM field must proactively address safety to build credibility and ensure patient wellbeing.

### 7.6 Clinical trial evidence for urate-lowering effects of TCM interventions

Several randomized controlled trials have evaluated the efficacy and safety of TCM-derived interventions in patients with hyperuricemia or related metabolic conditions. In a double-blind, placebo-controlled trial (NCT04144088), 60 patients with serum urate >8 mg/dL were randomized to Wu-Ling San, Yin Chen Wu-Ling San, or placebo for 4 weeks with 4 weeks of follow-up. After 8 weeks, the Yin Chen Wu-Ling San group showed a significantly lower serum urate level compared with placebo (8.1 vs. 9.1 mg/dL, p = 0.034), representing a 10.9% reduction from baseline. No serious adverse events were reported. Although the primary endpoint (serum urate <6 mg/dL at week 4) was not met, the results support the potential urate-lowering effect and favorable safety profile of this TCM formula (Leong et al., 2024).

In another randomized double-blind placebo-controlled trial (NCT04886297) involving 168 subjects with dyslipidemia, supplementation with resveratrol at 300 mg/d or 600 mg/d for 8 weeks significantly reduced serum uric acid levels in a dose-dependent manner compared with placebo (−23.60 ± 61.53 and −24.37 ± 64.24 μmol/L, respectively, p < 0.05), accompanied by a significant decrease in xanthine oxidase activity in the 600 mg/d group (p < 0.05). No serious adverse events were reported. These findings provide clinical evidence supporting the urate-lowering potential of resveratrol through XO inhibition (Zhou et al., 2023).

### 7.7 Future research priorities

Future research should address these critical gaps rather than perpetuate low-quality, descriptive studies. As highlighted by a recent evidence mapping study (Li et al., 2026), the methodological quality of existing systematic reviews on TCM for HUA is critically low, further underscoring the urgent need for well-designed, registered RCTs with rigorous reporting. First, pharmacokinetic studies should be integrated into early-phase investigations to establish clinically relevant dosing. Second, a systematic evaluation of the safety profile of botanical drugs—including potential renal and hepatic toxicity and interactions with conventional medications—is essential but currently lacking. Third, rigorous, double-blind, placebo-controlled RCTs with standardized extracts, predefined primary endpoints (e.g., sustained serum urate target achievement, gout flare reduction), and long-term follow-up are required. Fourth, microbial causality should be tested using FMT and germ-free models. Fifth, multi-omics and causal inference approaches (e.g., Mendelian randomization) should be employed to identify reproducible microbiota–metabolite–host signaling pathways.

In conclusion, TCM offers promising multi-target mechanisms for hyperuricemia that are mechanistically plausible and supported by a large body of preclinical evidence. However, the current evidence base suffers from significant methodological limitations, inconsistencies, and translational gaps. Addressing these shortcomings—through rigorous study design, proper blinding and randomization, pharmacokinetic characterization, safety assessment, and standardization—will be critical to transforming TCM from a traditionally described remedy into an evidence-based therapeutic option.
